# Draft Genome Sequence of Mycobacterium simiae, a Potential Pathogen Isolated from the Normal Human Oral Cavity

**DOI:** 10.1128/MRA.01185-20

**Published:** 2020-11-12

**Authors:** Varsha Chauhan, Kamal Shrivastava, Sakshi Anand, Chanchal Kumar, Anupriya Singh, Mandira Varma-Basil

**Affiliations:** a Department of Microbiology, Vallabhbhai Patel Chest Institute, University of Delhi, Delhi, India; University of Rochester School of Medicine and Dentistry

## Abstract

We report the draft genome sequence of Mycobacterium simiae, a slowly growing nontuberculous mycobacterium (NTM) isolated from a mouthwash sample of a healthy person. This genome of 6,603,693 bp exhibited a 66.13% GC content and 6,391 genes with 6,257 coding sequences, 3 rRNAs, and 78 tRNAs.

## ANNOUNCEMENT

Mycobacterium simiae is a slow-growing, photochromogenic, nontuberculous mycobacterium (NTM), phylogenetically related to 18 other species included in the M. simiae complex group, including *M. triplex,* M. genavense, *M. heidelbergense*, *M. lentiflavum*, *M. sherrisii*, *M. parmense*, *M. stomatepiae*, and *M. florentinum* (1, 2). M. simiae was first isolated in 1965 from rhesus monkey (3). This organism is ubiquitous and is found in environmental sources, including soil and water (4, 5). M. simiae also frequently colonizes the lung and is not always considered a potential pathogen in immunocompetent individuals (2, 6). However, several studies have reported disseminated multidrug-resistant M. simiae infection in both immunocompetent (7–11) and immunocompromised patients (12, 13). M. simiae infection has been associated with infection in lungs (8), parotid glands (7), the skeletal and genitourinary systems (9), and blood and bone marrow (12).

Here, we report the draft genome sequence of M. simiae isolated from the mouthwash sample of a healthy person from Delhi, India. The study was approved by the institutional ethical committee (IEC number VPCI/DIR/PS/IEC/2018). The mouthwash sample was decontaminated using 4% NaOH to prevent the growth of normal oral commensals. The sample was inoculated on Löwenstein-Jensen (LJ) medium and incubated at 37°C for 3 to 4 weeks (14). The isolated organism was confirmed as acid fast by Ziehl-Neelsen staining and further identified as an NTM by duplex PCR assay (15). The isolated NTM was identified as M. simiae by Hain Lifescience’s GenoType *Mycobacterium* CM/AS and by sequencing of the internal transcribed spacer (ITS) region. Whole-genome sequencing further confirmed the organism as M. simiae.

Genomic DNA from M. simiae was isolated using the cetyltrimethylammonium bromide (CTAB) DNA extraction method (16). Paired-end libraries were prepared using a NEBNext Ultra DNA library preparation kit. The sequencing was carried out on the Illumina Hi-Seq platform to generate 2 × 150-bp sequence reads at 100× sequencing depth. A total of 3,351,716 paired-end reads with an average length of 150 bp were recovered. The paired-end reads were quality controlled using FastQC v0.11.8 (17), filtered and trimmed by fastp v0.201 (18), and assembled with Shovill v1.0.4 (19). Default parameters were used for all software. A total of 3,256,162 paired-ends reads were obtained after filtering and trimming. Shovill assembled the reads in 123 contigs for a total assembly size of 6,603,693 bp with an *N*_50_ value of 189,271 bp. Annotation was carried out with Prokka v1.14.5 (20), which gave 6,259 coding sequences. The genome also carries 3 rRNAs and 78 tRNAs. The GC content of the genome is 66.13%. The average nucleotide identity (ANI) was calculated with DFAST taxonomic check (21). Mycobacterium sherrisii ATCC BAA-832 and Mycobacterium triplex DSM 44626 were the closest aligned sequenced genomes, with approximately 90% and 83.91% nucleotide identity, respectively. *M. triplex* is also a member of the M. simiae complex and known as a potential pathogenic species for human beings (1, 4).

The genome sequence of M. simiae presented here is from an isolate obtained from a mouthwash sample, representing essential data for future phylogenetic and comparative genome studies; thus, it will be useful for better understanding the M. simiae complex.

### Data availability.

The whole-genome sequence of M. simiae was deposited at GenBank under the BioProject accession number PRJNA647829 (SRA accession number SRR12285193).
